# Molecular Recognition of Biomolecules by Chiral CdSe Quantum Dots

**DOI:** 10.1038/srep24177

**Published:** 2016-04-11

**Authors:** Maria V. Mukhina, Ivan V. Korsakov, Vladimir G. Maslov, Finn Purcell-Milton, Joseph Govan, Alexander V. Baranov, Anatoly V. Fedorov, Yurii K. Gun’ko

**Affiliations:** 1 ITMO University, St. Petersburg, 197101, Russia; 2 School of Chemistry and CRANN, University of Dublin, Trinity College, Dublin 2, Ireland

## Abstract

Molecular recognition is one of the most important phenomena in Chemistry and Biology. Here we present a new way of enantiomeric molecular recognition using intrinsically chiral semiconductor nanocrystals as assays. Real-time confocal microscopy studies supported by circular dichroism spectroscopy data and theoretical modelling indicate an ability of left-handed molecules of cysteine and, to a smaller extent, histidine and arginine to discriminate between surfaces of left- and right-handed nanocrystals.

Most interactions between biomolecules involve molecular recognition, a natural mechanism that enables in biological systems such important processes as metabolism, immuno responses, information processing, and others. Receptors on biomolecules discriminate between left-handed (l) and right-handed (d) enantiomers of target molecules via their complementarity. This recognition occurs only if a target molecule fits energetically and structurally to a receptor molecule much like a key fits to a lock. It has been repeatedly demonstrated via experiment^1^,^2^,^3^,^4^,^5^ that a relatively small, but measurable difference in binding energy between heterocomplexes (d-l or l-d) and homocomplexes (d-d or l-l) of receptors and targets is sufficient for chiral recognition^6^. In the classical three-point interaction model^7^,^8^, strictly three points of attachment between receptor and target are needed to achieve the minimum of energy that is a necessary condition of complex formation. Although widespread, this simple geometrical model has recently had numerous exceptions in which three points of interaction were not required for chiral recognition to occur^5^,^9^.

Advances in approaches for obtaining chiral nanocrystals make it possible to imitate the molecular recognition processes at the nano/bio interface. Chiral nanocrystals can be formed using a range of conditions including employing semiconductor materials with a dissymmetric crystal lattice^10^, carrying out the synthesis in the presence of chiral reagents^11^,^12^,^13^,^14^,^15^,^16^ or via postsynthetic ligand exchange^17^,^18^,^19^,^20^,^21^. An approach^22^, tightly associated with chiral recognition on the surface of the nanocrystals, has been introduced most recently. Using enantioselective phase transfer technique, it has been shown that racemic mixtures (d:l = 50:50) of CdSe nanocrystal enantiomers naturally form during standard hot injection synthesis along with achiral nanocrystals and can be separated via an organic-aqueous phase transfer driven by chiral ligands. The presence of chiral defects (e.g. screw dislocations)^22^,^23^,^24^, which according to literature^25^,^26^,^27^,^28^,^29^ frequently appear during unidirectional growth of semiconductor nanocrystals, can result in the formation of nanocrystal enantiomers. Regardless of synthetic approach, chiral nanocrystals have a chiral pattern on their surface produced either by the presence of chiral ligands during the formation of the nanocrystals^11^ or by chiral defects affecting the entire volume of the nanocrystals and thus distorting their surface^22^. The chiral pattern on the surface of the chiral nanocrystals makes them excellent candidates for investigation of molecular recognition at the nano/bio interface.

Here we demonstrate further development of the enantioselective phase transfer technique and report a chiral recognition process between selected biomolecules and the surfaces of chiral nanocrystals. Through an interplay of Density Functional Theory (DFT) calculations, real-time photoluminescence (PL) measurements and Circular Dichroism (CD) spectroscopy data we observe that molecules of l-cysteine preferentially adsorb on the surface of d-nanocrystals since the formation of d-l heterocomplexes is energetically favoured over the formation of l-l homocomplexes.

To check if the molecules of cysteine can enantioselectively interact with the chiral surfaces of the QDs during phase transfer in solution, we chose CdSe QDs synthesized via a standard hot injection procedure and capped with achiral oleic acid. It has been shown in our previous paper that as-prepared ensembles of these nanocrystals form a racemic mixture of intrinsically chiral d- and l-nanocrystals and can be isolated in different phases by using a chiral phase transfer approach^22^. Therefore, since observation of molecular recognition at the nano/bio interface requires an enantioenriched sample of the QDs capped with achiral ligands, we used a two-step procedure of preliminary preparation of our samples: (1) separation of the nanocrystals enantiomers, (2) substitution of the chiral ligands with achiral ones. Then these samples were used for the molecular recognition study.

At the first step, as-prepared racemic mixtures of the CdSe QDs were separated via chiral phase transfer driven by l- and d-cysteine (the details are given in Methods in Supplementary Information, page S3). Figure 1A shows CD and absorption spectra recorded for the enantioenriched fractions of cysteine-capped nanocrystals in water. Both samples reveal an almost identical absorption curves with the first excitonic peak at 572 nm. CD spectra of l- and d-nanocrystals display near mirror image bands in the region corresponding to the absorption band, where d/l is the dextrorotatory/levorotatory CdSe nanocrystals. Here we use the d/l system to refer to the optical activity of the nanocrystals instead of the d/l system, since we do not have data on the actual configuration of each nanocrystal enantiomer. It is important to note that, as appears in the experimental data shown in Fig. 1A, dextrorotatory nanocrystals attached l-cysteine and vice versa. In other words, formation of d-l and l-d heterocomplexes is preferable during chiral phase transfer.

At the second step, a reverse phase transfer from water to chloroform (see Methods in Supplementary Information, page S3) was carried out using dodecanethiol (DDT) to substitute achiral molecules of DDT for the chiral molecules of cysteine on the surface of the QDs. The substitution of cysteine was confirmed with FTIR spectroscopy (see Supplementary Figure 1). As seen from Fig. 1B, both the d-l and l-d samples display a 10-nm blue shift of the absorption band due to the attachment of thiol ligands and corresponding shift of the CD bands. Also CD intensity normalized to optical density decreases eight times after removal of the chiral ligands, and only two the strongest CD bands corresponding to the first excitonic peak are distinguishable in the spectra. The intensity of these CD signals can be ascribed as intrinsic since the CD enhancement induced by the presence of chiral ligands on the QDs’ surfaces observed in the first step does not contribute to the CD spectra at this step.

If molecular recognition of cysteine on the QD surface promotes the separation of the nanocrystal enantiomers during the chiral phase transfer, the heterocomplexes and homocomplexes should form with different efficiency. At the third step, we compared the extent of complexing reactions for the heterocomplexes d-l and homocomplexes l-l of the QDs and l-cysteine. In this experiment we used the enantioenriched ensembles of the DDT-capped QDs from the second step, the concentrations and volumes of QDs solutions were equalized before the phase transfer (for details see Methods in Supplementary Information, page S3).

The extent of a reaction is given by 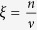, where *n* is the amount of reactant in moles and *ν* is the stoichiometric number of the reactant. The ratio *ξ*_*d*−l_/*ξ*_*l*−l_ can be estimated from *ξ*_*d*−l_/*ξ*_*l*−l_ = *n*_*d*−l_/*n*_*l*−l_ = *D*_*d*−l_/*D*_*l*−l_ under assumption that the stoichiometric numbers and the extinction coefficients of the reactants, as well as volumes of the solutions are the same (here *D* is optical density at *λ* = 554 nm of the QDs aqueous phase after chiral phase transfer).

In the experiments using CdSe QDs, the value of *ξ*_*d*−l_/*ξ*_*l*−l_ lies between 2.75 and 49 for ligand concentrations between 0.25 and 0.16 M indicating a preference of d-l heterocomplex formation. The CD and absorption spectra of the d- and l-QDs transferred to water after the chiral phase transfers using 0.16 M of l-cysteine are presented in Fig. 1C. In this case, the optical density of the l-l sample is very low (~0.02 at *λ* = 554 nm) in comparison to the d-l sample whose optical density is 0.48 and position of the first excitonic peak is the same as for the chloroform solution from step 2. Incomplete substitution of DDT molecules due to the low cysteine concentration used in step 3 is the most likely reason for the first excitonic peak not shifting back to the position corresponding to full capping with cysteine. Similarly, the CD bands of l-l sample are not distinguishable, and the d-l sample displays an increase of CD signal induced by the attachment of chiral ligands.

Aiming to demonstrate that recognition of cysteine is particular case of more general phenomenon of chiral recognition of biomolecules on chiral surfaces of CdSe QDs, we used histidine and arginine instead of cysteine at the third step (for details see Methods in Supplementary Information, page S3). As expected, difference in the extents of complexing reactions for d-l heterocomplexes and l-l homocomplexes of the QDs and L-histidine or l-arginine was observed, and the value of *ξ*_*d*−l_/*ξ*_*l*−l_ calculated for these complexes lies between 0.46 and 0.7 in the case of histidine and between 0.54 and 0.73 in the case of arginine for ligand concentration between 0.12 and 0.06 M. The data obtained indicate a preference of l-l homocomplexes formation. Histidine and arginine appear to be less selective target molecules compared to cysteine, probably because of missing thiol group.

To check how the presence of chiral ligands/stabilizer molecules during the synthesis procedure influences the ability of the QDs to discriminate between left-handed and right-handed molecules during postsynthetic chiral phase transfer, we used CdS QDs prepared by microwave induced heating with D- and L-enantiomeric forms of penicillamine as stabilizers. The CdS samples were prepared similarly to the intrinsically chiral CdSe samples via the reverse phase transfer from water to chloroform with achiral DDT molecules. It was expected that chiral molecules of the initial stabilizer penicillamine would be substituted with DDT. However, FTIR data shown in Supplementary Figure 1 indicate that the substitution occurred only partially. Supplementary Figure 2 shows CD spectra recorded for the CdS samples after the reverse phase transfer with DDT and after following comparison of the complexing reactions extents for d-l heterocomplexes and l-l homocomplexes of QDs and l-cysteine. The data obtained indicates the preference of *d* − l heterocomplexes formation similarly to the case of the intrinsically chiral samples. Most likely, incomplete substitution is the reason for small *ξ*_*d*−l_/*ξ*_*l*−l_ values found to be in the range of 1.24—1.83 for a ligand concentration in the range of 0.08–0.05 M, respectively.

CD spectroscopy allows only the obtaining of data on overall results of the molecular recognition process, however, real-time dynamics of the process and its detailed mechanisms remain unavailable for investigation using this technique. Therefore, we used confocal luminescent microscopy to monitor the molecular recognition of l-cysteine molecules on the chiral surfaces of the nanocrystals. Such monitoring was possible because the PL intensity of nanocrystals alters significantly during the substitution of ligands.

Enantioenriched ensembles of highly luminescent CdSe/CdS dots-in-rods capped with achiral DDT molecules and displayed high values of the intrinsic CD and CPL (spectra are shown in Supplementary Information Figure 4A) were used for the real-time dynamics experiments. For the reaction of chiral ligand exchange, ensembles of l- and d- CdSe/CdS nanocrystals deposited on glass substrates were treated with methanol to remove the DDT ligands and then with 0.01 M solution of l-cysteine in methanol to initiate chiral complexing reactions between cysteine and the nanocrystals surfaces. Stationary spectroscopy data shows (see Supplementary Information Figure 4B) that the PL intensity of the nanocrystals decreased typically by up to 30% after methanol treatment. Further treatment with l-cysteine solution resulted in more than a 60% decrease in the PL intensity of d-nanocrystals while the PL intensity of l-nanocrystals decreased only by 5%. According to the previously published data^30^, in the case of the QDs prepared using hot injection synthesis, exchange from hydrophobic to hydrophilic ligands generally leads to quenching of the nanocrystals luminescence. Therefore, the decrease in PL intensity observed by us in the complexing reactions can be used to estimate the extent of complexing reactions and so, to measure the value of 
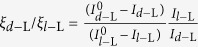
, where *I*^0^ and *I* are the PL intensities before and after the reaction^31^.

Using confocal microscopy with 0.97 s time resolution, we obtained time trajectories of the nanocrystals PL intensity during the l-cysteine recognition on the chiral surfaces of d- and l- CdSe/CdS nanocrystals (experimental details are given in Supplementary Information, page S7). Excluding intensity spikes observed to appear on both d-l and l-l curves at the moments immediately following the cysteine injections and most probably caused by inhomogeneous filling of the reaction chamber with cysteine molecules, both d-l heterocomplexes and l-l homocomplexes of the nanocrystals and l-cysteine displayed an overall decrease of the PL intensity, corresponding representative curves are shown in Fig. 2 (see also Supplementary Movies 1 and 2). The values of *ξ*_*d*−l_/*ξ*_*l*−l_ determined after approximately 450 s of reaction was found to be in the range of 19.1–24 that, in agreement with stationary CD spectroscopy data for solutions, indicates the preference of *d* − L heterocomplexes formation.

To support experimental finding, we performed DFT calculations for complexes of Cd_13_Se_13_ nanoclusters and molecule of l-cysteine. The calculations were carried out using the GAMESS program^32^ with the Hay-Wadt valence basis with pseudopotential^33^ and the B3LYP functional^34^.

Chiral discrimination of a target molecule by a receptor is possible only if both interacting moieties are chiral. To model chiral CdSe nanocluster, we distorted an ideal crystal structure of the cluster with a screw dislocation. Dislocation was introduced to the nanocluster as shown in Fig. 3A in one of close-packed 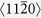 directions in {0001} planes, since 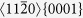 slip system is predominant for CdSe HCP crystal^35^. Magnitude of the Burger’s vector was calculated as 
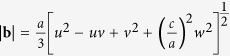
, where *a* and *c* are the constants of the CdSe crystal lattice, and *u, v, w* are Miller-Bravais indices. Magnitude of the vector was found to be equal to 1.43 Å. The displacements Δ_*z*_ applied to the atoms were given by 
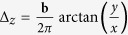
, where *x, y, z* are coordinates of the atoms along *X, Y, Z* axes. Oppositely distorted nanoclusters were obtained by the introduction of screw dislocations with negative (−**b**) and positive (**+b**) Burger’s vectors, representing the right-handed and left-handed nanoclusters, respectively. Top and side views of the distorted nanoclusters are shown in Figs 3B and 3C.

Molecular recognition of cysteine on the surface of the nanocrystals requires difference in binding energy of chiral molecules of cysteine to the oppositely distorted surface of the nanoclusters. To verify if the chirally distorted surface of CdSe nanocluster is enantioselective, we performed DFT calculations of the binding energy of l-cysteine and d- and l-distorted Cd_13_Se_13_ nanoclusters. For the calculation we attached a molecule of l-cysteine via three points to end-surfaces of the nanoclusters as shown in Figs 3B and 3C. The binding energy of l-cysteine and Cd_13_Se_13_ nanocluster was calculated by taking the difference between the total energy of the l-cys-l-Cd_13_Se_13_ or l-cys-d-Cd_13_eS_13_ complexes with the optimized geometry and the sum of the total energy of the Cd_13_Se_13_ nanocluster with the same geometry as in the complexes and the total energy of l-cysteine with optimized geometry. Atoms of Cd_13_Se_13_ nanocluster were fixed during optimization. The difference between binding energies of the l-cys-d-Cd_13_Se_13_ complex and the l-cys-l-Cd_13_Se_13_ complex was found to be equal to 198 meV (for details see Supplementary Information, page S9), indicating the possibility of chiral molecular recognition in the interface between CdSe nanocluster and cysteine. This result is in line with the previous reports for cysteine on the surface of distorted ZnS nanoclusters^22^ and chiral kinks on gold surfaces^4^,^5^.

We have investigated the molecular recognition of l-cysteine molecules on the chiral surfaces of the CdSe and CdS nanocrystals. Using a chiral phase transfer technique^22^, we have verified an ability of nanocrystal enantiomers to discriminate between left-handed and right-handed molecules of cysteine during the postsynthetic chiral phase transfer. In CD experiments, formation of d-l heterocomplexes of the nanocrystals with molecules of l-cysteine has been found to be up to 50 times more preferable comparing to the formation of l-l homocomplexes. Analogous experiments performed with L-histidine and L-arginine have showed a preference of l-l homocomplexes formation. Enantioselectivity of the nanocrystals surfaces has also been confirmed by real-time PL intensity dynamics acquired during the reaction of l-cysteine recognition. Analysis of intensity trajectories for d-l and l-l complexing reactions has revealed a preference of d-l heterocomplexes formation, what is in agreement with CD spectroscopy. Experimental findings were supported by DFT calculations predicting that l-cysteine molecules bind to the surface of (+)-distorted CdSe nanocluster 198 meV weaker then to the surface of (−)-distorted nanocluster.

## Additional Information

**How to cite this article**: Mukhina, M. V. *et al*. Molecular Recognition of Biomolecules by Chiral CdSe Quantum Dots. *Sci. Rep.* **6**, 24177; doi: 10.1038/srep24177 (2016).

## Supplementary Material

Supplementary Information

Supplementary Movie 1

Supplementary Movie 2

## Figures and Tables

**Figure 1:**
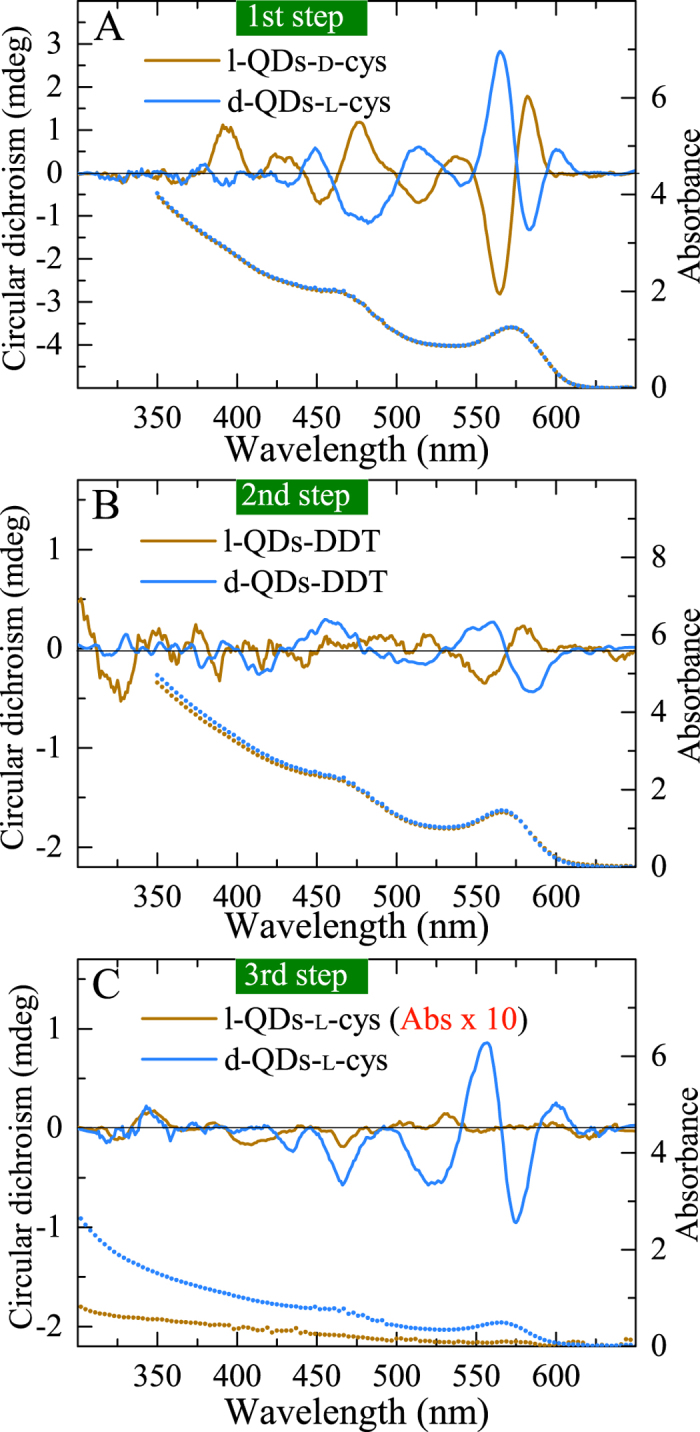
Preparation for (**A,B**) and observation of (**C**) the molecular recognition of l-cysteine on the chiral surfaces of the CdSe QDs. The absorption (dotted lines) and CD (solid lines) spectra of the enantioenriched solutions of the CdSe QDs after the chiral separation with l- and d-cysteine in water (**A**), after the substitution of chiral cysteines with achiral DDT in chloroform (**B**), and after the comparison of the chiral phase transfer efficiency for d-l against l-l complexes of the nanocrystals and cysteine (**C**).

**Figure 2:**
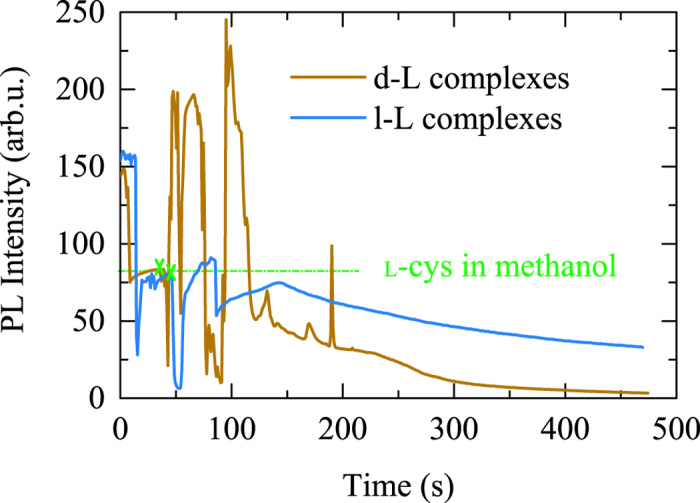
Representative dependences of PL intensity on time for the reactions of l-cysteine recognition on the chiral surfaces of d- and l- CdSe/CdS nanocrystals. Green marks show the moments of reaction beginning. Green dotted line shows a zero level of intensity achieved after non-enantioselective treatment with methanol.

**Figure 3:**
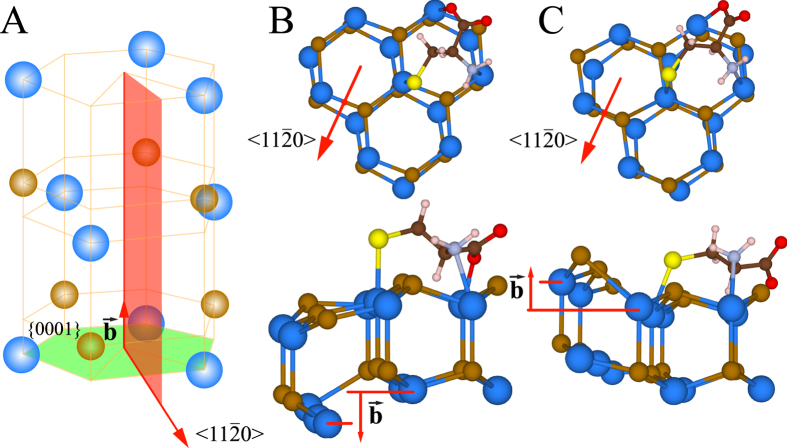
(**A**) Basal slip system 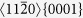 in crystal lattice of CdSe. (**B,C**) Top (top) and side (bottom) views of atomistic models of Cd_13_Se_13_ nanoclusters with right (**B**) and left (**C**) screw dislocations. Atomistic models were prepared using Vesta software^36^. Red dots indicate cores of dislocations.
