# Supplementary material for: Molecular Recognition of Biomolecules by Chiral CdSe Quantum Dots
Source: Sci Rep. 2016 Apr 11;6:24177. doi: 10.1038/srep24177 (PMC4827062; doi:10.1038/srep24177)
Supplement: Supplementary Information [file srep24177-s1.pdf]

Supplementary information

Molecular Recognition of Biomolecules by Chiral CdSe Quantum Dots

Maria V. Mukhina[*,†], Ivan V. Korsakov[†], Vladimir G. Maslov[†], Finn Purcell Milton[‡], Joseph Govan[‡], Alexander V. Baranov[†], Anatoly V. Fedorov[†], and Yurii K. Gun'ko[*,†,‡]

[†]ITMO University, 197101 St. Petersburg, Russia, and [‡]School of Chemistry and CRANN, University of Dublin, Trinity College, Dublin 2, Ireland

[*]To whom correspondence should be addressed: mmuxina@gmail.com; igounko@tcd.ie

Materials

CdSe oleic acid stabilized quantum dots

This synthesis was carried out using a modified method from literature [1]. Se-TBP solution preparation: 0.1105 g (1.399 mmol) of Se was mixed with and 3.0 ml of TBP under an argon atmosphere and sonicated until the Se become completely dissolved , indicated by a clear transparent solution. CdSe Synthesis: 0.50 g (0.0039 mol) of CdO, 4.0 g of oleic acid and 10.0 g of ODE (octadecene) were weighed into a 100 ml 3-neck round-bottom flask equipped with a condenser and thermometer. Following this, the flask was put under vacuum and degassed for 20 minutes at 30 °C. After this, the flask was switched to argon; the temperature was increased to 260 °C and allowed to stabilize. Time was then allowed for the CdO to convert to Cd $(oleate)_2$ which was indicated by a colour change from brown to colourless. At this point, the Se-TBP solution was injected. Growth was allowed to proceed for a set amount of time, 30 seconds to produce the size tested in our experiment. The reaction was then removed from the heating mantle and allowed to cool to 200 °C, followed by injection of 20 ml of degassed acetone to precipitate the quantum dots (QDs). The resulting QD solution was cleaned using a number of dissolution and precipitation cycles with dry hexane and HPLC grade acetone.

CdS penicillamine stabilized quantum dots

CdS penicillamine stabilized quantum dots were synthesized using a microwave synthesis. All materials used were from Sigma Aldrich and used as it, all water used was derived from a Millipore machine (18.2 MΩ. Into a 200mL microwave vessel was placed water (40mL Millipore), Penicillamine solution (10mL 0.01M), NaOH (2M, 300uL, series 1 and 500uL series 2), $CdCl_2$ solution (0.01M, 8mL) and thioacetamide (0.01M, 2mL). The vessel was then placed in a Starr series Microwave and was heated for 1 minute in a reflux apparatus. The samples were then transferred to an appropriate vessel, covered in aluminium foil and stored for further work-up. The samples were reduced in volume under reduced pressure and then filtered via ultracentrifugation using nanogap filters with a 30kDa cut-off. The samples were washed repeatedly via ultracentrifugation with water and then dispersed in Millipore water and stored at 4 ºC.

CdSe/CdS core sell quantum dots-in-rods

For synthesis of CdSe/CdS core-shell quantum dots-in-rods, n-octadecylphosphonic acid (ODPA) capped QDs were prepared as described in [2]. At the next step, for QRs 0.120 g of S was dissolved in 1.5 ml of TOP under argon. To this was added 8 x 10^{-8} mol of ODPA capped CdSe QDs in TOP solution. 0.057 g of CdO, 3 g TOPO, 0.29 g ODPA and 0.08 g n-hexylphosphonic acid (HPA) were added to a 3-neck flask. Initially the mixture was heated to 150 ˚C under argon, after which the solution was placed under vacuum and degassed for 30 minutes. The flask was then flushed with argon and heated to 300 ˚C, holding at this temperature until the solution turned optically clear and colourless, indicating formation of the Cd complex. Following this, 1.5 g of TOP was injected into the flask and the solution was heated further to 360 ˚C. This was then followed by injection of the S-TOP and CdSe QDs stock solution. The quantum dots-in-rods are then allowed to grow for 8 minutes after which the heating mantle is removed. After the solution cooled to below 150 ˚C, 20 ml of degassed methanol was injected to

precipitate the quantum dots-in-rods and the solution was allowed to cool to room temperature. The quantum dots-in-rods were fully separated using centrifugation and then re-dispersed in chloroform.

Methods

Step 1: chiral phase transfer.

For the optically enriched samples, 200 µl of nanocrystals stock solution were re-dissolved in 1 mL of chloroform. The solutions obtained were cooled for 15 min at 4 °C to slow the process. Then 5 vol % of 0.25 M methanol solution of D- or L-cysteine was added to the nanocrystals solution followed by stirring. After 1−2 min of stereospecific solubilization, 1 mL of distilled water with pH 10−11 was added to the solution. pH was varied by the addition of aqueous solution of KOH. To initiate the phase transfer, the mixture was vigorously shaken and left for 1−2 min. After complete separation, the aqueous phase was collected for further analysis.

Step 2: reverse phase transfer.

For phase transfer from water to chloroform [3], 1 mL of dodecanthiol (DDT) and 2 mL of acetone were added to 1 mL of aqueous solution of cysteine capped nanocrystals obtained after the enantioselective phase transfer. To initiate the phase transfer, the mixture was vigorously shaken and heated to 56 °C. After a few minutes the mixture were centrifuged, washed few times with toluene and methanol, and then the nanocrystals capped with DDT were re-dissolved in chloroform.

Step 3: comparison of the extents of d-L and l-L complexing reactions via chiral phase transfer.

For comparison of the extents of d-L and l-L complexing reactions, 1 ml of 10^{-5} M chloroform solutions of d- or l-nanocrystals were cooled for 15 min at 4 °C to slow the process. Then 5 vol % of methanol solution of L-cysteine, L-histidine or L-arginine were added to the nanocrystals solution followed by stirring. Concentration of the ligand solutions used for the phase transfer lied between 0.16 and 0.25 M for cysteine and between 0.06 and 0.12 M for histidine and arginine. After 1−2 min of stereospecific solubilization, 1 mL of distilled water with pH 10−11 or pH 6-7 was added to the solution in the case of using cysteine and histidine or arginine, respectively. pH was varied by the addition of aqueous solution of KOH or HCl. To initiate the phase transfer, the mixture was vigorously shaken and left for 1−2 min. After complete separation, the aqueous phase was collected for further analysis. In the case of using histidine and arginine, the aqueous phase was re-dispersed in isopropanol.

CD and CPL measurements

The spectra of circular dichroism and absorption were studied using a Jasco J-1500 spectrometer. The spectra of circularly polarized luminescence and luminescence were studied using a Jasco CPL-300 spectrometer.

FTIR data on CdSe QDs

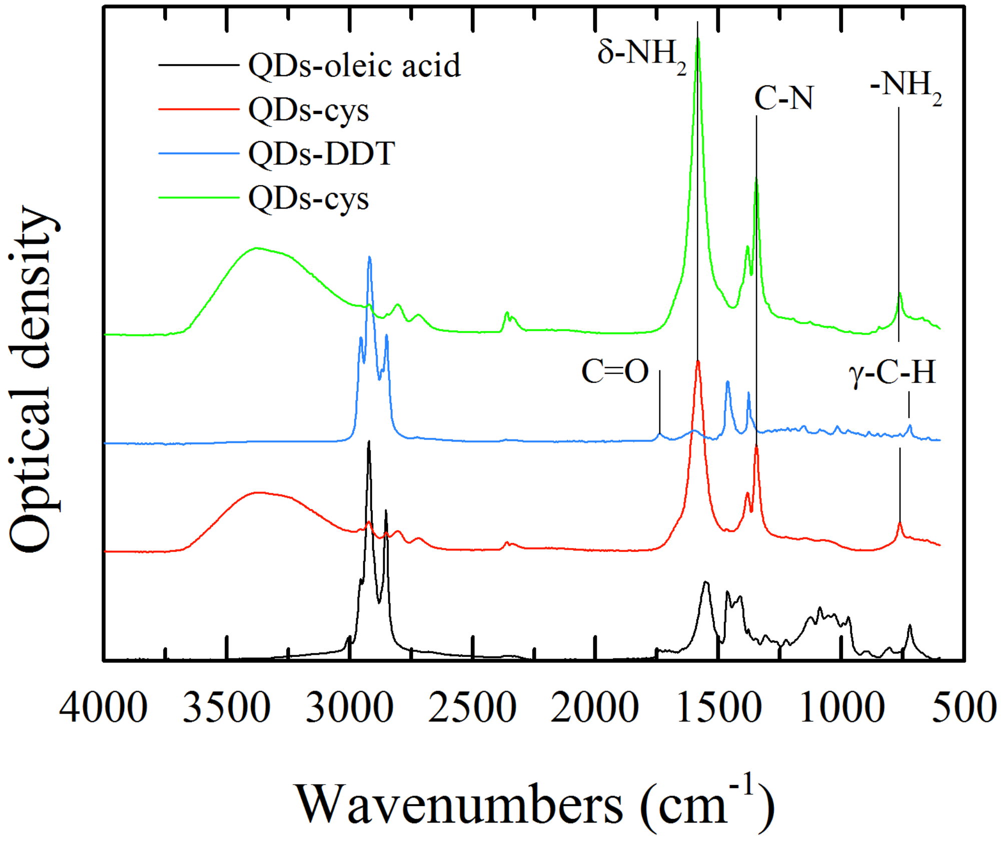

Supplementary Figure 1. FTIR spectra of the CdSe QD samples from the bottom to the top: in initial solution in chloroform; in water after the enantioselective phase transfer with cysteine; in chloroform after the reverse phase transfer of the aqueous phase from water to the chloroform with achiral ligand dodecanethiol (DDT); again in water after the enantioselective phase transfer with cysteine.

FTIR data analysis

The substitution of oleic acid with cysteine (red curve) is confirmed by an appearance of the sharp and very intensive bands of amino group around 760 and 1580 cm^{-1}. Following substitution of cysteine with DDT (blue curve) is confirmed by an absence of any of bands of amino group (760, 1580, 3400-3500 and 3200 cm^{-1}). The band C=O (1740 cm^{-1}) can be ascribed to acetone that was used during the reverse phase transfer. Weak band around 1600 can be ascribed not to small traces of amino groups of cysteine, but to toluene used for washing the nanocrystals after the reverse phase transfer. The presence of toluene is supported by γ-C-H band at 730 cm^{-1}, which is characteristic for mono-substituted aromatics compounds.

CD spectra of CdS QDs

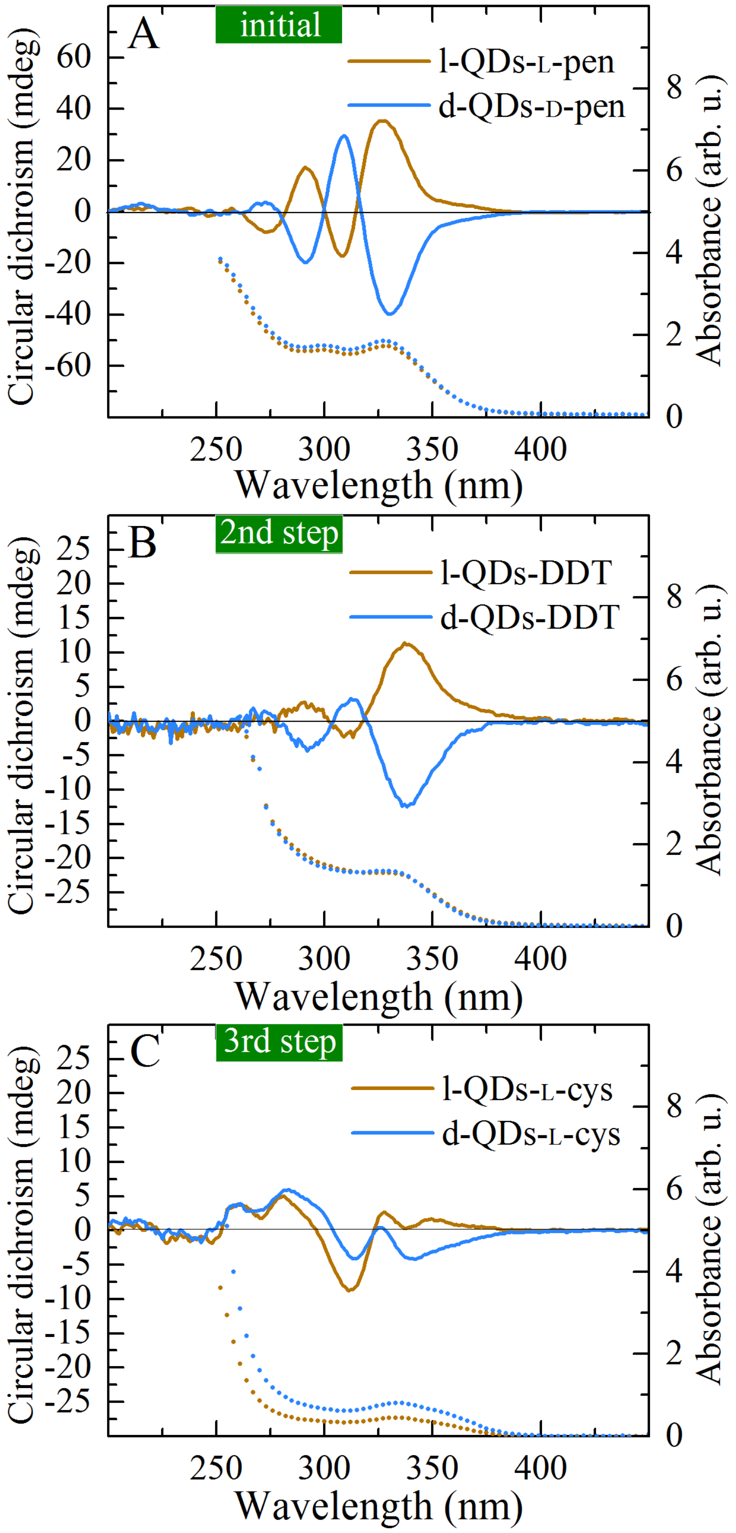

Supplementary Figure 2. Preparation for (B) and observation of (C) the molecular recognition of L-cysteine on the chiral surface of CdS QDs. The absorption (dotted lines) and CD (solid lines) spectra of the enantioenriched solutions of the CdS QDs after the synthesis in the presence of L- and D -penicillamine in water (A), after the substitution of chiral penicillamine with achiral DDT in chloroform (B), and after the comparison of the chiral phase transfer efficiency for d-L against l-L complexes of the nanocrystals and cysteine (C). The CdS samples were prepared

similarly to the intrinsically chiral CdSe samples via the reverse phase transfer from water to chloroform with achiral DDT molecules.

FTIR data on CdS QDs

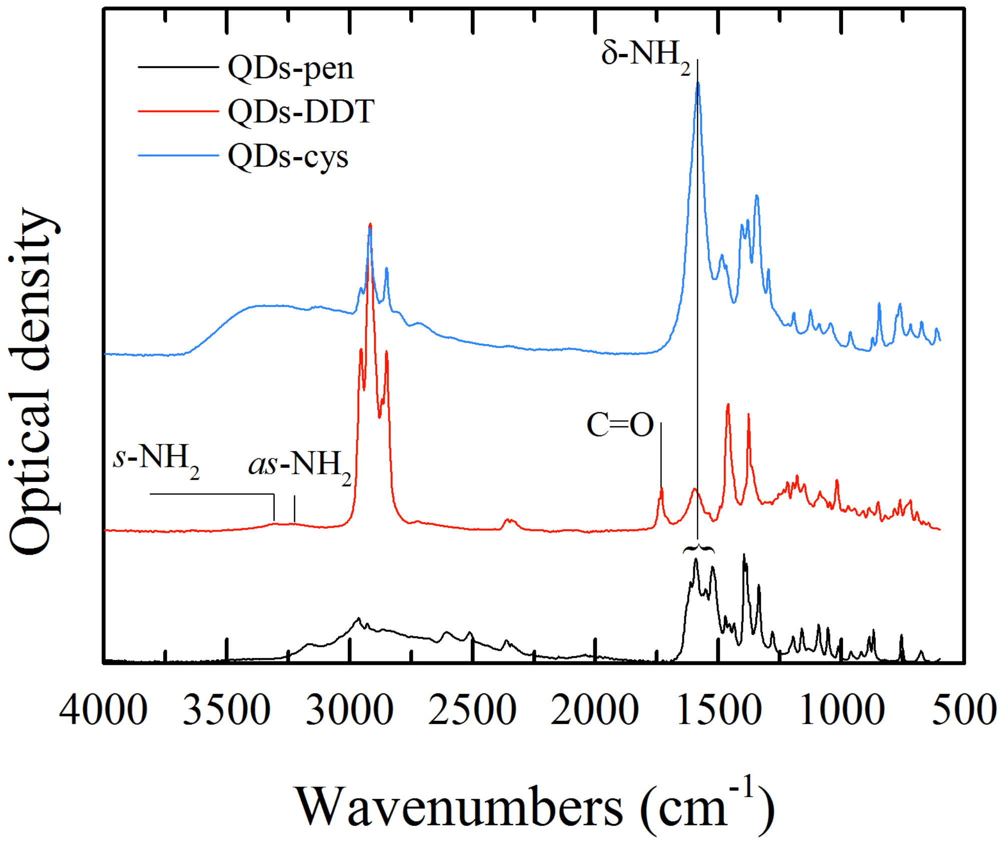

Supplementary Figure 3. FTIR spectra of the CdS QD samples from the bottom to the top: in initial solution in water capped with penicillamine; in chloroform after the reverse phase transfer from water to chloroform with the achiral ligand dodecanethiol (DDT); again in water after the enantioselective phase transfer with cysteine.

FTIR data analysis

FTIR spectra do not confirm the substitution of penicillamine with DDT (red curve) since after the reverse phase transfer the st-NH_2 bands of amino group at 3300 and 3220 cm^{-1} are present in the spectra.

Studies of real-time dynamics of the molecular recognition process

The complexing reactions of L-cysteine and the nanocrystals during molecular recognition process were studied using confocal microscopy. Enantioenriched ensembles of highly luminescent and intrinsically chiral CdSe/CdS nanocrystals (characterization data are shown on page S8 of Supplementary Information) capped with achiral DDT molecules and displayed a high CD values were used in this experiment. Enantiomeric enrichment with L- or D-nanocrystals was achieved via a standard two step procedure described on page S3 of Supplementary Information: enantioselective phase transfer for separation of the nanocrystals enantiomers followed by the reverse phase transfer for substitution of the chiral ligands with achiral ones.

Typically, 5 μl of the nanocrystal solution with concentration 10^{-6} M were deposited on the bottom of a chamber similar to ones usually used as hemocytometers described everywhere. Dynamics experiments were performed for solid layer of the nanocrystals dried on substrate. The depth of the chamber was 0.17 mm and its volume was 70 μl. The chamber were used to ensure a constant level of liquid during the experiment and, thus, to avoid random defocusing of the microscope.

The reactions were recorded as time series of 2D luminescent maps of the samples. The luminescent maps were acquired on a Zeiss LSM 710 confocal laser scanning luminescent microscope equipped with a spectrometer. Time series were recorded with 63x objective from 7.65 μm x 7.65 μm area with 0.97 s integration time per frame. The reagents (methanol and solution of L -cysteine in methanol) necessary to the experiment were supplied to the reaction chamber using two syringes securely attached to the inlet of the reaction chamber.

The experiment was started by adding 50 μl of methanol to the reaction chamber. Addition of methanol caused 40-45 % PL intensity decreasing, partly because of hydrophobic ligands removal caused by the presence of methanol and partly because of defocusing. Since the level of liquid did not alter after methanol addition, focus stayed the same during all further measurements. Therefore, constant level of PL intensity obtained after methanol addition could be taken as a zero level for molecular recognition reaction. It could be taken also that all further PL intensity alterations were induced by the interactions of chiral molecules of cysteine with chiral surfaces of the nanocrystals. To initiate the complexing reaction, 20 μl of 0.01 M solution of L -cysteine in methanol was added to the reaction chamber.

Records of the complexing reactions of L-cysteine and D- and L-nanocrystals are available as separate video files Supplementary Movies 1 and 2, respectfully.

CdSe/CdS dots-in-rods characterization

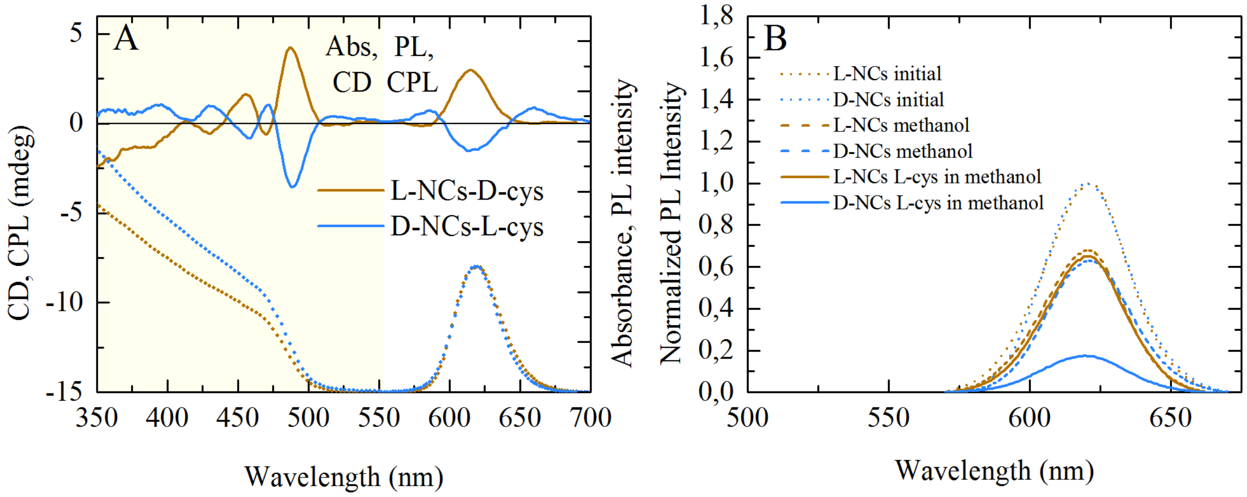

Supplementary Information Figure 4. (A) The absorption, PL (dotted lines), CD, and CPL (solid lines) spectra of the enantioenriched solutions of the CdSe/CdS quantum dots-in-rods after the chiral separation with L- and D-cysteine in water. (B) Molecular recognition of L-cysteine molecules on chiral surfaces of the CdSe/CdS quantum dots-in-rods deposited on glass substrate. PL spectra of the enantioenriched ensembles of the nanocrystals (NCs) capped with achiral DDT molecules (dotted lines); the NCs ensembles after the treatment with methanol (dashed lines); the NCs ensembles after the chiral complexing reactions with 0.01 M L-cysteine solution in methanol. PL spectra were normalized to initial PL intensity. The absorption, PL, CD, and CPL spectra were recorded using CD and CPL spectrometers (Jasco). Spectra after treatments with methanol and L-cysteine solution in methanol (Fig. SI 4 B) were recorded 5 min later after the treatments with reagents.

DFT Calculations

	$E_{complex}$ (a.u.)	$E_{cluster}$ (a.u.)	$E_{binding}$ (a.u.)	$E_{binding}$ (eV)
Undistorted L-cys-$Cd_{13}Se_{13}$ complex	-1317.5285	-984.7979	-0.1697	-4.6167
L-cys-(+)$Cd_{13}Se_{13}$ complex distorted by dislocation +1.43 A	-1317.4644	-984.7423	-0.1612	-4.3865
L-cys-(-)$Cd_{13}Se_{13}$ complex distorted by dislocation -1.43 A	-1317.4629	-984.7336	-0.1685	-4.5851

The binding energy of L-cysteine and nanocluster was calculated as

$E_{binding} = E_{complex} - E_{cluster} - E_{cys}$,

where $E_{complex}$ is the total energy of the L-cys-(+)$Cd_{13}Se_{13}$ complex or L-cys-(−)$Cd_{13}Se_{13}$ complex, $E_{cluster}$ is the total energy of the $Cd_{13}Se_{13}$ nanocluster with the same geometry as in the complexes, and E_{cys} is the total energy of L-cysteine molecule. The total energy of L-cysteine molecule with optimized geometry was calculated to be -332.560841909 a.u.

Legends of video files available as Supplementary Information

Supplementary Movie 1: Records of the complexing reactions of L-cysteine and D-nanocrystals

Supplementary Movie 2: Records of the complexing reactions of L-cysteine and L-nanocrystals

References

(1) Bullen, C. R.; Mulvaney, P. Nucleation and Growth Kinetics of CdSe Nanocrystals in Octadecene. *Nano Lett*. **4**, 2303-2307 (2004).

(2) Carbone, L.; Nobile, C.; De Giorgi, M.; Sala, F. D.; Morello, G.; Pompa, P.; Hytch, M.; Snoeck, E.; Fiore, A.; Franchini, I. R.; Nadasan, M.; Silvestre, A. F.; Chiodo, L.; Kudera, S.; Cingolani, R.; Krahne, R.; Manna, L. Synthesis and micrometer-scale assembly of colloidal CdSe/CdS nanorods prepared by a seeded growth approach. *Nano Lett.* **7**, 2942-2950 (2007).

(3) Gaponik, N.; Talapin, D. V.; Rogach, A. L.; Eychmüller, A.; Weller, H. Efficient phase transfer of luminescent thiol-capped nanocrystals: from water to nonpolar organic solvents. *Nano Lett*. **2**, 803−806 (2002).
